# Dendritic Cell-Based Approaches in the Fight Against Diseases

**DOI:** 10.3389/fimmu.2014.00078

**Published:** 2014-02-26

**Authors:** Rafael Freitas-Silva, Maria Carolina Brelaz-de-Castro, Valéria Rêgo Pereira

**Affiliations:** ^1^Department of Natural Sciences, University of Pernambuco, Garanhuns, Brazil; ^2^Department of Immunology, Aggeu Magalhães Research Center, Oswaldo Cruz Foundation, Recife, Brazil

**Keywords:** dendritic cells, immunotherapy, vaccine, cancer, infectious diseases

## Dendritic Cell Role in the Immune System and Its Manipulation

The immune system works to contain infections through activation of different molecules and cell types. Correct presentation of antigens by antigen-presenting cells (APCs) is a critical step necessary to initiate an immune response. APCs have the ability to take-up and process antigens, and express high levels of co-stimulatory and major histocompatibility complex (MHC) molecules bound to antigens (1).

Dendritic cells (DCs) are innate immune cells first characterized and reported by Ralph Steinman in 1973 (2). For their unique properties and features, DCs are the most important APCs acting at the interface of innate and adaptive immunity, which results in the activation of immune responses in the body. Distinct subsets of DCs are associated with lineage and receptor expression patterns (3).

Dendritic cells have different roles in the immune system, such as activation and regulation of adaptive immune responses, and other opposing functions in the induction of tolerance and anergy (4). During immune responses, DCs are crucial decision makers toward the development of naïve T cells to T helper type 1 (Th1) or type (Th2) profile (5).

Among the different families of molecules expressed by DC to aid in their function, one of them is the family of toll-like receptors (TLR). TLR which are expressed by different types of DCs, and bind to common molecules associated with pathogens. Once bound, molecules such as bacterial lipopolysaccharide and hypomethylated CpG DNA, can induce activation of biochemical cell pathways, resulting in overexpression of MHC, co-stimulatory molecules (CD80, CD86), and cytokines (6).

In this context, a number of methods have been available to manipulate DCs from diverse sites in the body resulting in activated cells for therapy. These methods include reinfusion of unloaded DCs; reinfusion of DCs co-cultured with peptides or proteins of interest; *in vivo* DC loading; DC transfection with antigen-encoding viruses or nucleic acids; and DC-derived exosomes (7, 8). After this, DCs might be ready to promote protection or treat specific diseases.

In this context, the availability of methods to manipulate DCs in laboratory, arise as an important tool for immunointerventions in different diseases. In this opinion article, we focused on the basis of DC approaches already available in the field of cancer currently in test for infectious diseases, and future interventions that are needed.

## Dendritic Cell Approaches for Cancer

Since initial tests with murine models, activated DCs have been an attractive alternative to treat cancer as an immunostimulatory vaccine. This vaccine has the ability to induce effective cancer immunity by inducing Th1 cells and specific cytotoxic T lymphocytes to tumor antigens, as well as natural killer (NK) cells (9, 10). The potential of anti-cancer vaccines also lies on their capacity to stimulate long-lasting memory T cells against tumor antigens. Among the subsets of memory T cells, the presence of central memory (T_cm_) cells has been associated with a better antitumor function than effector memory cells (11).

The first attempt of vaccination was performed with DCs derived from patients with non-Hodgkin’s lymphoma who have failed current treatment. Immunoglobulin idiotype from the patient’s tumor were used to load DCs *ex vivo* and then were reinjected into the patient’s body – what resulted in the complete remission of the tumor (12).

To date, many clinical assays employing different methods to activate DCs have been in test for different types of cancers. Most trials were performed using autologous DCs pulsed *ex vivo* with tumor antigens or derived peptides, and administered to patients with or without chemotherapy or other immune agent (13). However, other types of interventions are in course in clinical trials, such as those using DCs engineered to express tumor antigens with or without molecules such as CD40 ligand, CD70, and TLR-4 (14, 15). Important results were shown in one trial performed by Tel et al. (16), who reported a strong immune-specific response against melanoma after administration of a particular subset of DCs, called plasmacytoid-DCs (pDCs) pulsed with melanoma specific antigens. pDCs have been seen as interesting players in this task, since once properly activated they are able to produce high levels of gamma-interferon (IFN-γ) and elicit a robust Th1 immune response.

Most clinical assays have used *ex vivo* manipulation of patient’s peripheral blood monocytes cultured in the presence of interleukin (IL)-4 and recombinant granulocyte macrophage-colony stimulating factor (GM-CSF) to achieve DCs for therapy (17). In this way, a DC-based preparation of autologous cells expanded *ex vivo* in the presence of a prostatic acid phosphatase/GM-CSF fusion protein (sipuleucel-T, Provenge^®^) was approved by the US FDA and other international regulatory agencies for use in patients with advanced metastatic prostate cancer (18). From trials initiated in 2012, sipuleucel-T is involved in at least seven trials against prostate cancer, combining sipuleucel-T with: different regimens of radiotherapy (19); administration of monoclonal antibody against cytotoxic T lymphocyte-associated protein 4 (CTLA-4) (20); administration of recombinant human IL-7 (21); and injection of DNA-based anti-cancer vaccine together with GM-CSF (22). Thus, it is expected that further results with sipuleucel-T will be disclosed in the next years.

However, *ex vivo* manipulation of DCs are limited by some factors, such as the high cost, the long time needed to handled in laboratory, and ultimately the high risk of infection to the patients (23, 24). The latter issue is clearly one of the most important, since cancer patients might be already immunocompromised and susceptible to diverse pathogen infections.

To overcome this issue, searching for new alternatives to *ex vivo* manipulation are in course, and many of them are being developed, such as activation and loading DCs with antigens *in vivo*. One good example is the use of specific peptides combined with GM-CSF to attract and activate DCs *in vivo*, which showed prospective clinical results (25). Other strategy is the use of cancer cells genetically modified to express GM-CSF, resulting in the attraction and activation of DCs (26). Another tactic is the delivery of oncolytic viruses, which preferentially infect and kill cancer cells (27).

One of the most promising approaches is the *in vivo* targeting of specific DC receptors using antibodies coupled with antigens (28, 29). It was verified that administration of this type of vaccine with DC activators such as TLR3, TLR-7-8, and CD40 agonists allows the establishment of immunity in diverse diseases settings, including infections [e.g., malaria and human immunodeficiency virus (HIV)] and cancer (30, 31).

Although prospective results are disclosed and expected, most clinical trials fail to go beyond Phase II due to a reduced success rate. This indicates that more studies are needed to fill gaps in the comprehension of the immune response necessary to eliminate cancer and explore this knowledge in DC cancer vaccines, such as the use of TLR agonists and the particular role of each DC subset. In parallel, work groups are dedicating efforts to identify better correlates of clinical efficacy to evaluate results from clinical trials more properly.

## Dendritic Cell Approaches for Infectious Diseases

Dendritic cell manipulation offers an interesting approach to fight against infectious diseases, and an alternative to prompt a protective immunity, since some treatments are ineffective or inexistent in those (32, 33). Previous studies have shown that DCs can induce protection against different pathogens, including protozoan, bacteria, and virus. DCs recognize microorganisms through TLR or C-type lectin receptors (34, 35). Vaccination works have reported protection against leishmaniasis (36, 37), Herpes simplex virus (38, 39), influenza virus (40), and *Candida albicans* (41), among other pathogens, such as HIV.

Human immunodeficiency virus has different mechanisms of evasion from the immune system, and nowadays the main source of treatment to infected patients is to follow combination antiretroviral therapy (cART) for life. However, attention was drawn to promising results obtained by the use of DC-based vaccine against HIV. Lu et al. (42) performed the first success clinical trial described, and found a significant reduction in plasma viral load (VL) after administration of autologous DCs loaded with inactivated autologous virus in HIV-1 infected patients. At least 12 studies have achieved interesting results, and evolved to clinical trials with HIV-1 infected patients and reported HIV-1 specific-immunological responses (43). Recently, García et al. (44) observed a significant decrease in VL in HIV-1 chronic infected patients who have interrupted cART treated with autologous monocyte-derived DCs pulsed with autologous heat-inactivated whole HIV. Previously, García et al. (45) also showed promising results with significant drop in VL in HIV-1 infected patients off-cART treated with the same vaccine preparation. Based on this, it is expected that in the next few years good results will be achieved, enhancing the chances to develop an immunointervention that could help infected individuals.

Although now it is possible to target vaccine antigens to DCs in T and B areas and to modulate their function with adjuvants, there is still no currently approved DC therapy for infectious diseases, and most experimental approaches are especially with animal models (46). One good example is leishmaniasis, which is one of the most important neglected diseases that cause deaths and morbidity in more than 88 countries. Current human anti-leishmania vaccines available are limited by their inefficiency to confer protection against the different species and also by their safety, which is contested. DCs approaches for leishmaniasis were proposed by different groups of research with remarkable results showing low levels of parasite burden and high levels of Th1 cytokines in animals treated (47, 48). However, results from studies with animal models might be difficult to translate the results to humans, and it will remain a goal for further investigations. DCs therapy for leishmaniasis and other infectious diseases would aid mainly refractory patients to current treatments due to high toxic drugs that are available for use or the increasing number of resistant pathogens. Furthermore, immunocompromised individuals, such as those with AIDS or grafted, would be benefited by more safety and effective treatments against different pathogens.

## Conclusion

In the last couple of years, DC therapies approaches have been shown to be feasible and secure. Successful results were and are being obtained with cancer patients and animal models. DCs have an extraordinary capacity to orchestrate the host’s immune response, which offers new perspectives for the development of vaccines and immunotherapeutic strategies against cancer and infectious diseases among others. However, due to the success that is been observed with cancer and also due to the efforts that is being put by many research groups in the development of antigens and adjuvants with good immunological stimulatory capacities, we believe that in a closer future DC therapies will be also a viable approach to treat and/or prevent infectious diseases.
